# Antioxidant and Anti-Inflammatory Effects of 3-Dehydroxyceanothetric Acid 2-Methyl Ester Isolated from *Ziziphus jujuba* Mill. against Cisplatin-Induced Kidney Epithelial Cell Death

**DOI:** 10.3390/biom11111614

**Published:** 2021-10-31

**Authors:** Dahae Lee, Kyo Bin Kang, Gwi Seo Hwang, You-Kyoung Choi, Tae Kon Kim, Ki Sung Kang

**Affiliations:** 1 College of Korean Medicine, Gachon University, Seongnam 13120, Korea; pjsldh@gachon.ac.kr (D.L.); seoul@gachon.ac.kr (G.S.H.); kosmos@gachon.ac.kr (Y.-K.C.); 2 College of Pharmacy, Sookmyung Women’s University, Seoul 04310, Korea; kbkang@sookmyung.ac.kr; 3 College of Science & Engineering, Jungwon University, Chungbuk 28024, Korea

**Keywords:** *Ziziphus jujuba* Mill., LLC-PK1, nephrotoxicity, oxidative stress, inflammation

## Abstract

Cisplatin is a platinum-based chemotherapeutic agent for treating solid tumors; however, it presents a risk factor for nephropathy. In the present study, we investigated the antioxidant and anti-inflammatory effects of 3-dehydroxyceanothetric acid 2-methyl ester (3DC2ME) isolated from *Ziziphus jujuba* Mill. in LLC-PK1 cells following cisplatin-induced cytotoxicity. These cells were exposed to 3DC2ME for 2 h, followed by treatment with cisplatin for 24 h. The treated cells were subjected to cell viability analysis using the Ez-Cytox assay. Reactive oxygen species (ROS) were detected via 2′, 7′- dichlorodihydrofluorescein diacetate (DCFH-DA) staining. In addition, western blotting and fluorescent immunostaining were performed to evaluate protein expressions related to oxidative stress and inflammation pathways. Pretreatment with 3DC2ME protected LLC-PK1 cells from cisplatin-induced cytotoxicity and oxidative stress. In addition, pretreatment with 3DC2ME upregulated heme oxygenase 1 (HO-1) via the nuclear factor erythroid 2-related factor 2 (Nrf2) pathway in the cisplatin-treated LLC-PK1 cells. Furthermore, the increase in the expressions of IκB kinase α/β (IKKα/β), inhibitor of kappa B alpha (IκBα), nuclear factor kappa B (NF-κB), inducible nitric oxide synthase (iNOS), and cyclooxygenase-2 (COX-2) in these cells was inhibited. These results provide basic scientific evidence for understanding the antioxidant and anti-inflammatory effects of 3DC2ME isolated from *Z. jujuba* against cisplatin-induced kidney epithelial cell death.

## 1. Introduction

The kidneys perform a variety of pivotal functions, such as the control of fluids and solutes, metabolic waste excretion, endocrine function, and blood pressure control, as well as drug metabolism and excretion [1]. Therefore, the kidneys are a major target for various drug-induced toxicities. In particular, cisplatin-induced nephrotoxicity remains an important global medical problem [2]. However, cisplatin is still widely prescribed in clinical practice, despite the high prevalence of cisplatin-induced nephrotoxicity (34.1%) [3]. Cisplatin induces nephrotoxicity and exerts toxic effects through one or more cellular mechanisms. In cisplatin-induced nephrotoxicity, elevated oxidative stress and decreased antioxidant enzymes can destroy cell organelle structures and interfere with cellular processes. Oxidative stress has been linked to apoptosis, inflammation, and mitochondrial DNA damage and enhances the secretion of tumor necrosis factor-α (TNF-α), which amplifies the inflammatory response [4]. Various in vitro and in vivo studies have shown that antioxidants and anti-inflammatory agents isolated from natural products exhibit protective effects against cisplatin-induced nephrotoxicity [5,6].

However, to date, there have been no clinically approved natural product-based drugs for cisplatin-induced nephrotoxicity. In two previous studies, we isolated ceanothane- and lupine-type triterpenoids from the roots of jujubes (*Ziziphus jujuba*, Rhamnaceae). Among the triterpenoids, 3-dehydroxyceanothetric acid 2-methyl ester (3DC2ME, see Supplementary Material) was effective at inhibiting cisplatin-induced renal cell toxicity by modulating cellular molecular pathways involving mitogen-activated protein kinase (MAPK), apoptosis, and autophagy in kidney epithelial LLC-PK1 cells [7,8]. It has been reported that root extracts of *Z. jujuba* exhibit antioxidant and anti-inflammatory effects and are thus effective against carbon tetrachloride-induced hepatic damage and diabetic neuropathy [9,10]. Its antioxidant and anti-inflammatory actions were expected to have a positive effect on cisplatin-induced renal cell toxicity. In particular, in our previous study, we reported the efficacy of 3DC2ME against cisplatin-induced toxicity in LLC-PK1 cells at a lower concentration than N-acetyl cysteine, an antioxidant, thus postulating the possibility of 3DC2ME having an antioxidant effect [8]. In addition, multiple targets related to MAPK and the apoptosis pathway support the system-level mechanism of 3DC2ME [7]. Until recently, MAPK, apoptosis, autophagy, oxidative stress, and inflammation have been known to be important mechanisms related to cisplatin-induced renal cell toxicity [5,6]. This study provides further evidence, with an emphasis on oxidative stress and inflammation, for the protective effect of 3DC2ME against cisplatin-induced kidney epithelial cell death.

## 2. Materials and Methods

### 2.1. Cell Culture and Cell Viability Assay

Porcine proximal tubular epithelial cells (LLC-PK1) were obtained from the American Type Culture Collection (ATCC, Manassas, VA, USA) and grown as a monolayer culture in a 55 cm^2^ culture dish in an incubator containing 5% carbon dioxide and 95% oxygen at 37 °C. Dulbecco’s Modified Eagle’s Medium (DMEM, GIBCO, Grand Island, NY, USA) was supplemented with 1% penicillin/streptomycin (GIBCO, Grand Island, NY, USA) and 10% fetal bovine serum (FBS, GIBCO, Grand Island, NY, USA) and used as the culture medium. The LLC-PK1 cells were placed on a 96-well clear flat-bottom plate and pre-treated with 3DC2ME for 2 h. After subsequent treatment with 25 μM cisplatin for 24 h, the cell viability was determined using Ez-Cytox reagents (Daeil Lab Service Co., Seoul, Korea), according to the manufacturer’s instructions [11]. Representative cell images were visualized using an IX50 fluorescence microscope.

### 2.2. Measurement of Intracellular Reactive Oxygen Species

Intracellular reactive oxygen species (ROS) were determined using a cell-permeable fluorescent indicator. The LLC-PK1 cells were placed on a black 96-well clear flat-bottom plate and pre-treated with 3DC2ME. After 2 h, the cells were treated with 25 μM cisplatin for 24 h and loaded with 10 μM 2′,7′-dichlorofluorescin diacetate (H2DCFDA, Sigma–Aldrich, St. Louis, MS, USA). After incubation in the dark for 30 min, the unreacted H2DCFDA was removed from the 96-well plate using phosphate-buffered saline (PBS, Sigma–Aldrich, St. Louis, MS, USA). The intensity of fluorescent DCF at 495/517 nm(ex/em) was measured using a fluorescent microplate reader (SPARK 10M). Representative cell images were obtained using an IX50 fluorescent microscope [12].

### 2.3. Fluorescent Immunostaining

The LLC-PK1 cells were placed on 8-well chamber slides and pre-treated with 3DC2ME. After 2 h, the cells were treated with 25 μM cisplatin for 24 h, fixed with 4% paraformaldehyde (Sigma–Aldrich, St. Louis, MS, USA) for 30 min, and washed with PBS. The cells permeabilized using 0.1% Triton X-100 (Sigma–Aldrich, St. Louis, MS, USA) in PBS (PBS-T) were washed with 0.01% PBS-T and blocked in 2% normal horse serum (NHS, Sigma–Aldrich, St. Louis, MS, USA) for 24 h. The cells were incubated with primary antibodies for nuclear factor erythroid 2-related factor 2 (Nrf2, Cell Signaling Technology, Inc., Beverly, MA, USA) and nuclear factor kappa B (NF-κB, Cell Signaling Technology, Inc., Beverly, MA, USA) overnight at 4 °C, followed by incubation with FITC-conjugated goat anti-rabbit IgG (Cell Signaling Technology, Inc., Beverly, MA, USA) overnight at 4 °C in the dark. After washing with PBS-T, the cover slips were mounted on slides with a ProLong^®^ Gold antifade reagent containing DAPI (Invitrogen Molecular Probes, Eugene, OR, USA). Representative cell images were obtained using an IX50 fluorescent microscope [13].

### 2.4. Protein Extraction

The LLC-PK1 cells were placed on 6-well culture plates and pre-treated with 3DC2ME. After 2 h, the cells were treated with 25 μM cisplatin for 24 h, and the harvested cell pellets were washed with ice-cold PBS. The cell pellets were lysed using radioimmunoprecipitation assay (RIPA) buffer (Cell Signaling Technology, Inc., Beverly, MA, USA) in the presence of 1 mM phenylmethylsulfonyl fluoride (PMSF, Sigma–Aldrich, St. Louis, MS, USA) and a 1 × EDTA-free protease inhibitor cocktail (Sigma–Aldrich, St. Louis, MS, USA) on ice for 20 min. The suspension was then centrifuged at 12,000 rpm for 20 min at 4 °C. The supernatants were harvested as the total protein and the cell pellets were suspended in a cytoplasmic extraction buffer containing 10 mM 4-(2-hydroxyethyl)-1-piperazineethanesulfonic acid (HEPES, Sigma–Aldrich, St. Louis, MS, USA), pH 7.9, 10 mM potassium chloride (Sigma–Aldrich, St. Louis, MS, USA), 0.1 mM ethylenediaminetetraacetic acid (Sigma–Aldrich, St. Louis, MS, USA), 0.1% tergitol (Sigma–Aldrich, St. Louis, MS, USA) in the presence of a 1 × EDTA-free protease inhibitor cocktail on ice for 15 min. The suspension was then centrifuged at 1000 rpm for 4 min at 4 °C. The supernatants were harvested as the cytosolic fraction, and the cell pellets were suspended in a RIPA buffer in the presence of 1 mM PMSF and a 1 × EDTA-free protease inhibitor cocktail on ice for 15 min. The suspension was then centrifuged at 13,000 rpm for 5 min at 4 °C. The supernatants were harvested as nuclear fractions [14].

### 2.5. Western Blotting Analysis

The same amount of protein from each of the total proteins, cytoplasmic extractions, and nuclear fractions was transferred to a polyvinylidene difluoride transfer membrane from a precast 4–15% Mini-PROTEAN TGX gel (Bio-Rad, Hercules, CA, USA). The membranes were exposed to primary antibodies, including Nrf2, heme oxygenase 1 (HO-1), phospho-IκB kinase α/β (p-IKKα/β), IκB kinase α (IKKα), IκB kinase β (IKKβ), phospho-inhibitor of kappa B alpha (p-IκBα), inhibitor of kappa B alpha (IκBα), nuclear factor kappa B (NF-κB), inducible nitric oxide synthase (iNOS), cyclooxygenase-2 (COX-2), lamin B, and GAPDH, followed by horseradish peroxidase-conjugated secondary antibodies. All the antibodies were purchased from Cell Signaling Technology, Inc. (Beverly, MA, USA). The detection was performed using ECL Advance Western Blotting Detection Reagents (GE Healthcare, Buckinghamshire, UK) and a Fusion Solo Chemiluminescence System (PEQLAB Biotechnologie GmbH, Erlangen, Germany). The signal intensity of each band was quantified using Fusion-Capt v16.10 software (PEQLAB Biotechnologie GmbH).

### 2.6. Statistical Analysis

The statistical analysis was conducted using analysis of variance (ANOVA) followed by a multiple comparison test with a Bonferroni adjustment. The analysis was performed using SPSS ver. 19.0 software (SPSS Inc., Chicago, IL, USA). All the assays were performed in triplicate and were repeated at least thrice. Statistical significance was set at *p* values of less than 0.05.

## 3. Results

### 3.1. Time-Dependent Effect of Cisplatin on Oxidative Stress in LLC-PK1 Cells

**Figure 1:**
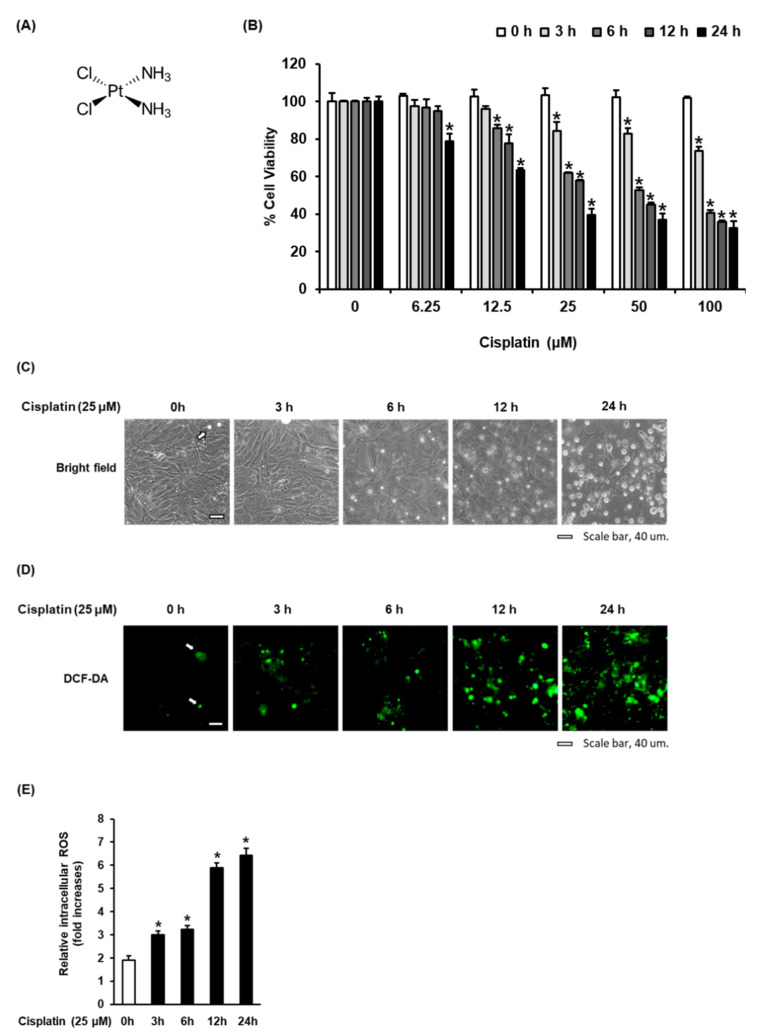
Time-dependent cisplatin-induced oxidative stress in LLC-PK1 cells. (**A**) Chemical structure of cisplatin. (**B**) Cell viability as determined using the Ez-Cytox assay after treatment with 25 M cisplatin for 3, 6, 12, and 24 h. (**C**) Representative images obtained using an IX50 fluorescent microscope. The white arrows indicate dead cells (scale bar = 40 μm). (**D**) LLC-PK1 cells were treated with 25 M cisplatin for 3, 6, 12, and 24 h, and stained with H2DCFDA. Representative fluorescent images obtained using an IX50 fluorescent microscope. The white arrows indicate intracellular ROS (scale bar = 40 μm). (**E**) The fold-increases in intracellular ROS levels are represented as a bar graph. Control cells were treated with the vehicle only (mean ± SD, * *p* < 0.05 compared to the control).

We investigated the cytotoxicity of cisplatin in LLC-PK1 cells. Cisplatin (Figure 1A) inhibited the viability of LLC-PK1 cells in a concentration- and time-dependent manner. After treatment with 25 μM cisplatin for 24 h, the cell viability decreased to 39.41 ± 3.27% (Figure 1B), with morphological changes such as loss of cell adhesion and blebbing (Figure 1C). In agreement with these results, the levels of reactive oxygen species (ROS) visualized as green H2DCFDA fluorescence were significantly increased in a time-dependent manner after treatment with 25 µM cisplatin (Figure 1D). Treatment with 25 µM cisplatin for 24 h resulted in a 6.48 ± 0.37-fold increase in intracellular ROS (Figure 1E). These results suggest that the cytotoxicity of cisplatin in LLC-PK1 cells was related to an increase in intracellular ROS.

### 3.2. Effect of 3DC2ME on Cisplatin-Induced Oxidative Stress in LLC-PK1 Cells

**Figure 2:**
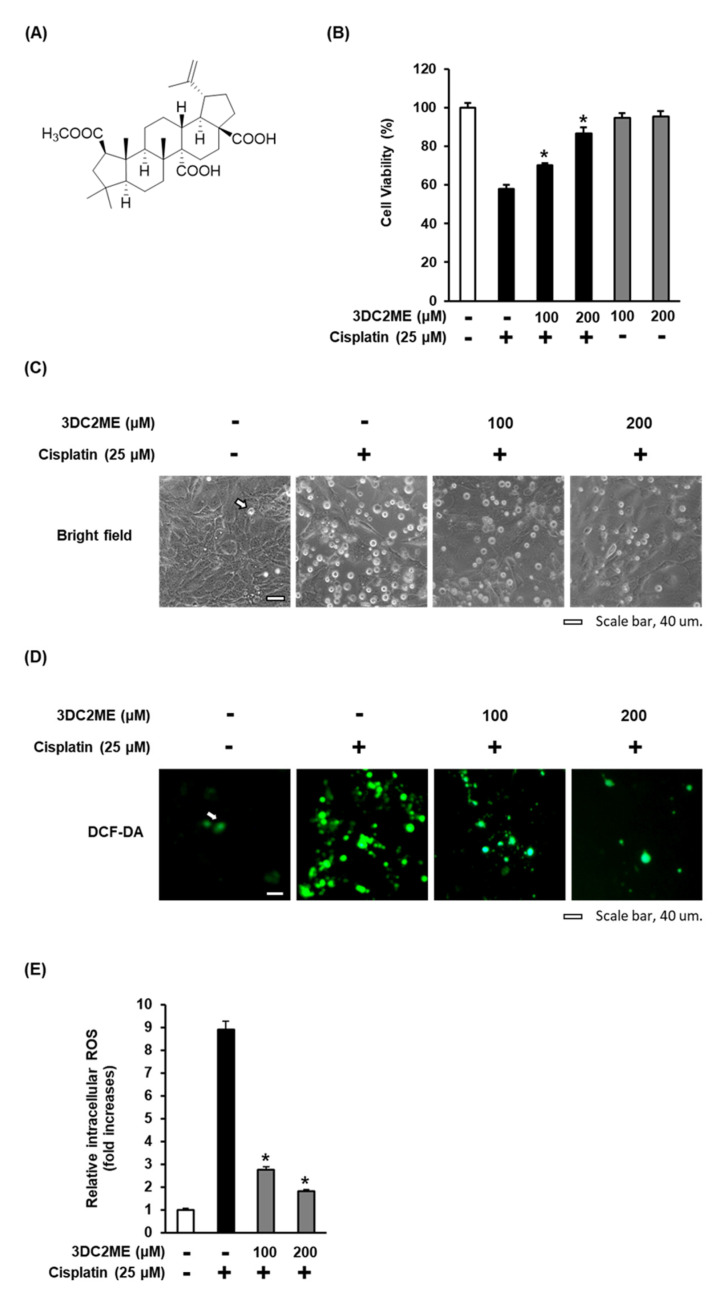
Effect of 3DC2ME on cisplatin-induced oxidative stress in LLC-PK1 cells. (**A**) Chemical structure of 3DC2ME. (**B**) Cell viability as determined using the Ez-Cytox assay after treatment with 25 µM cisplatin in the presence or absence of 100 or 200 µM 3DC2ME for 24 h. (**C**) Representative images obtained using an IX50 fluorescent microscope. The white arrows indicate dead cells (scale bar = 40 μm). (**D**) LLC-PK1 cells exposed to 25 μM cisplatin in the presence or absence of 100 or 200 µM 3DC2ME for 24 h and stained with H2DCFDA. Representative fluorescent images were obtained using an IX50 fluorescent microscope. The white arrows indicate intracellular ROS (scale bar = 40 μm). (**E**) Fluorescent intensity of DCF determined using a fluorescent microplate reader. The fold-increases in intracellular reactive oxygen species (ROS) levels are represented as a bar graph. Control cells were treated with the vehicle only (mean ± SD, * *p* < 0.05 cisplatin-treated LLC-PK1 cells).

To further investigate the protective effect of 3DC2ME (Figure 2A) against cisplatin-induced cytotoxicity and oxidative stress in the LLC-PK1 cells, the cells were treated with 25 μM cisplatin in the presence or absence of 100 or 200 µM 3DC2ME for 24 h. After treatment with 25 µM cisplatin, the viability of the cells was significantly reduced to 57.96 ± 3.94% compared with the control cells treated with the vehicle only, with morphological changes such as loss of cell adhesion and blebbing, whereas these effects were reversed following treatment with 100 and 200 µM 3DC2ME. After pre-treatment with 100 and 200 µM 3DC2ME, the viability of the cells increased to 70.24 ± 1.12% and 86.53 ± 3.16%, respectively, compared with the cells treated with 25 μM cisplatin (Figure 2B,C). In agreement with these results, the increased levels of ROS, visualized as green H2DCFDA fluorescence after treatment with 25 µM cisplatin, were significantly reduced after pretreatment with 100 and 200 µM 3DC2ME (Figure 2D). Treatment with 25 µM cisplatin for 24 h resulted in an 8.89 ± 0.38-fold increase in intracellular ROS compared with control cells treated with vehicle only, whereas treatment with 100 and 200 µM 3DC2ME inhibited levels of intracellular ROS by 2.76 ± 0.13-fold and 1.81 ± 0.09-fold, respectively, compared with the cells treated with 25 μM cisplatin (Figure 2 E). These results suggest that pretreatment with 3DC2ME prevented cisplatin-induced cytotoxicity and oxidative stress.

### 3.3. Effects of 3DC2ME and/or Cisplatin on the Expression of Nrf2/HO-1 Proteins Associated with Antioxidant Pathways in LLC-PK1 Cells

**Figure 3:**
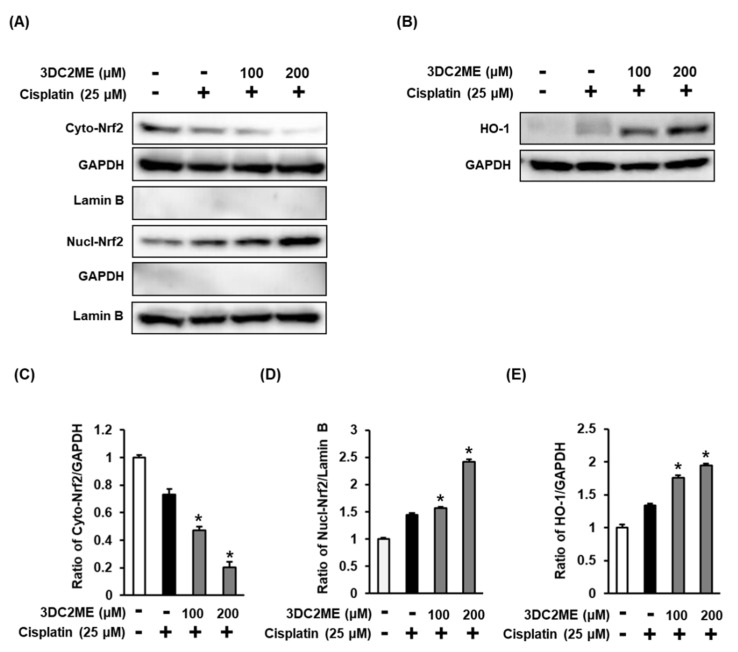
Effects of 3DC2ME and/or cisplatin on the expression of heme oxygenase 1 (HO-1)/nuclear factor erythroid 2-related factor 2 (Nrf2) proteins associated with antioxidant pathways in LLC-PK1 cells. (**A**,**B**) LLC-PK1 cells were exposed to 25 μM cisplatin in the presence or absence of 100 or 200 µM 3DC2ME, and western blot analysis was performed using antibodies for Nrf2, HO-1, lamin B, and GAPDH. (**C**–**E**) The ratios of nuclear Nrf2, cytosolic Nrf2, and HO-1 compared with the control cells are represented as bar graphs (mean ± SD, * *p* < 0.05 cisplatin-treated LLC-PK1 cells).

**Figure 4:**
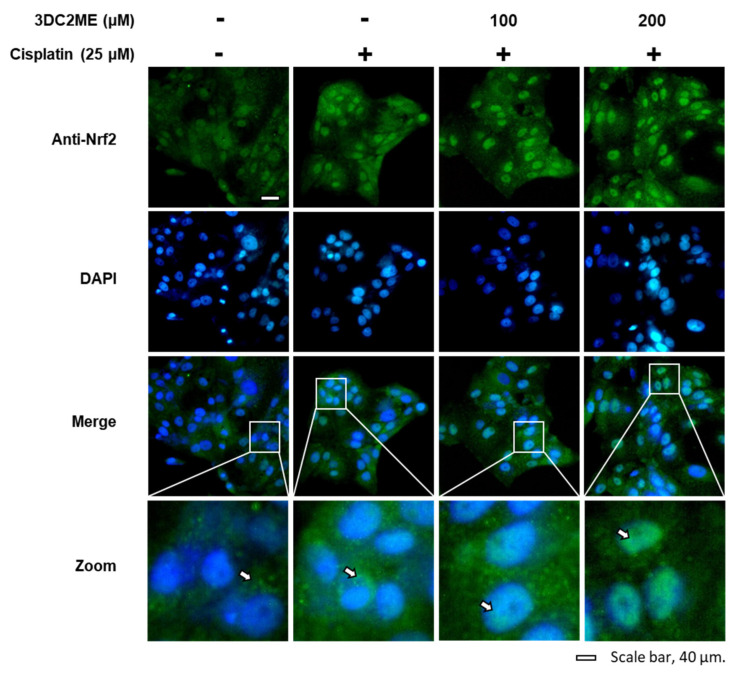
Effects of 3DC2ME and/or cisplatin on nuclear translocation of Nrf2 in LLC-PK1 cells. LLC-PK1 cells were exposed to 25 μM cisplatin in the presence or absence of 100 or 200 µM 3DC2ME, and immunofluorescent staining was performed using antibodies for Nrf2 that were visualized with a goat anti-rabbit IgG-heavy and light chain antibody FITC conjugate (green) and mounted with ProLong^®^ Gold antifade reagent with DAPI stained nucleus (DAPI). Representative images were visualized using an IX50 fluorescent microscope. The white arrows indicate the presence of Nrf2 (scale bar = 40 μm).

The role of Nrf2/HO-1 proteins associated with antioxidant pathways in the protective effect of 3DC2ME against cisplatin-induced cytotoxicity and oxidative stress in the LLC-PK1 cells was evaluated using western blotting. After treatment with 25 µM cisplatin, the ratios of nuclear Nrf2/lamin B and HO-1/GAPDH were increased, while that of cytoplasmic Nrf2/GAPDH was decreased, compared with the control cells treated with the vehicle only. However, the former ratios were further intensified after pre-treatment with 100 and 200 µM 3DC2ME compared with the cells treated with 25 μM cisplatin (Figure 3). In agreement with these results, the nuclear translocation of Nrf2 visualized with a goat anti-rabbit IgG-heavy and light chain antibody FITC-conjugate (green) was further intensified after pre-treatment with 100 and 200 µM 3DC2ME compared with the cells treated with 25 μM cisplatin (Figure 4). These results suggest that pre-treatment with 3DC2ME prevented cisplatin-induced cytotoxicity and oxidative stress via the induction of nuclear translocation of Nrf2-mediated HO-1 expression.

### 3.4. Effects of 3DC2ME and/or Cisplatin on the Expression of Proteins Associated with Inflammatory Pathways in LLC-PK1 Cells

**Figure 5:**
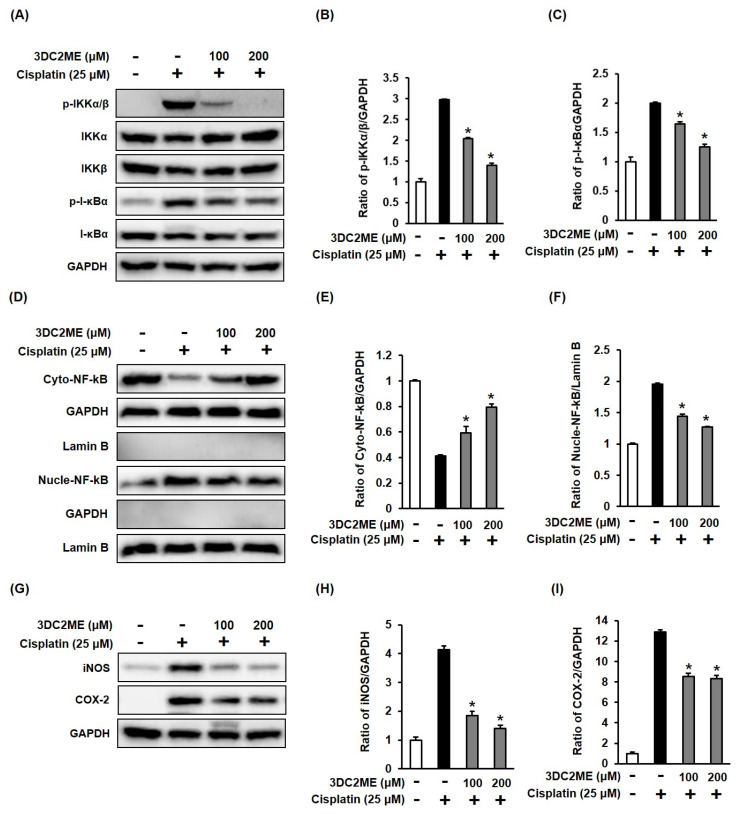
Effects of 3DC2ME and/or cisplatin on the expression of proteins associated with inflammatory pathways in LLC-PK1 cells. (**A**) LLC-PK1 cells were exposed to 25 μM cisplatin in the presence or absence of 100 or 200 µM 3DC2ME for 24 h, and western blot analysis was performed using antibodies for phospho-IκB kinase α/β (p-IKKα/β), IκB kinase α (IKKα), IκB kinase β (IKKβ), phospho-inhibitor of kappa B alpha (p-IκBα), inhibitor of kappa B alpha (IκBα), nuclear factor kappa B (NF-κB), and glyceraldehyde 3-phosphate dehydrogenase (GAPDH). (**B**,**C**) The ratios of p-IKKα/β and p-I-κBα compared with the control cells are represented as bar graphs. (**D**) LLC-PK1 cells were exposed to 25 μM cisplatin in the presence or absence of 100 or 200 µM 3DC2ME for 24 h, and western blot analysis was performed using antibodies for NF-kB, lamin B, and GAPDH. (**E**,**F**) The ratios of nuclear NF-kB and cytosolic NF-kB compared with the control cells are represented as bar graphs. (**G**) LLC-PK1 cells were exposed to 25 μM cisplatin in the presence or absence of 100 or 200 µM 3DC2ME for 24 h, and western blot analysis was performed using antibodies for inducible nitric oxide synthase (iNOS), cyclooxygenase-2 (COX-2), and GAPDH. (**H**,**I**) The ratios of iNOS and COX-2 compared with the control cells are represented as bar graphs (mean ± SD, * p < 0.05 cisplatin-treated LLC-PK1 cells).

**Figure 6:**
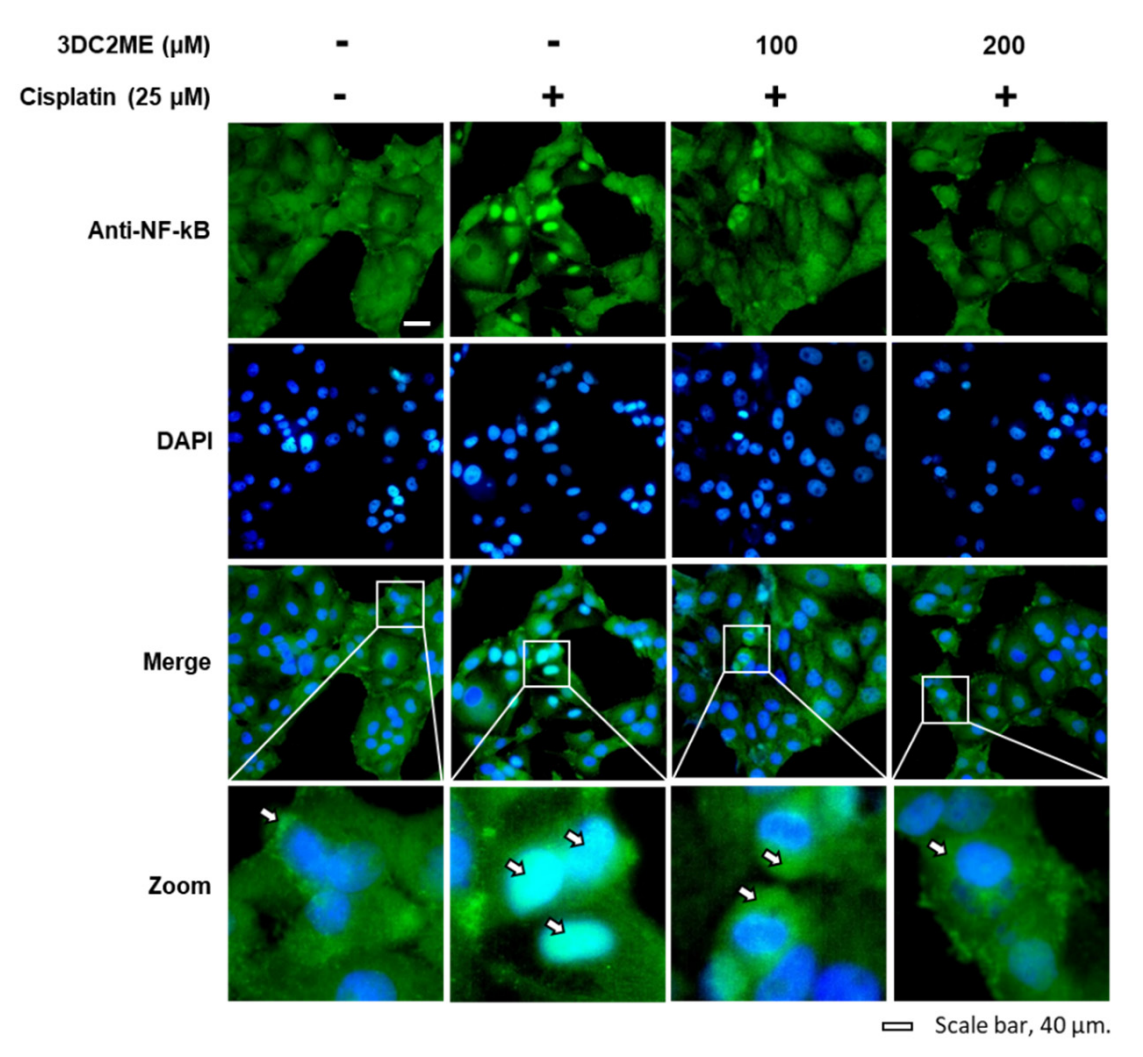
Effects of 3DC2ME and/or cisplatin on nuclear translocation of NF-kB in LLC-PK1 cells. LLC-PK1 cells were exposed to 25 μM cisplatin in the presence or absence of 100 or 200 µM 3DC2ME, and immunofluorescent staining was performed using antibodies for NF-kB that was visualized with a goat anti-rabbit IgG-heavy and light chain antibody FITC conjugate (green) and mounted with ProLong^®^ Gold antifade reagent with DAPI stained nucleus (DAPI). Representative images were obtained using an IX50 fluorescent microscope. The white arrows indicate the presence of NF-kB (scale bar = 40 μm).

The role of the protein expressions of IKKα/β, I-κBα, NF-kB, iNOS, and COX-2 associated with inflammatory pathways in the protective effect of 3DC2ME against cisplatin-induced cytotoxicity and oxidative stress in LLC-PK1 cells was evaluated using western blotting. The increased phosphorylation of IKKα/β and I-κBα after treatment with 25 µM cisplatin was inhibited following pre-treatment with 100 and 200 µM 3DC2ME (Figure 5A–C). In agreement with these results, the ratio of cytoplasmic NF-kB/GAPDH was reduced and the ratio of nuclear NF-kB/lamin B increased after treatment with 25 µM cisplatin compared with the control cells treated with the vehicle only, whereas these ratios were reduced after pretreatment with 3DC2ME (Figure 5D–F). In addition, the increased protein expression of iNOS and COX-2 after treatment with 25 µM cisplatin was reversed after pre-treatment with 100 and 200 µM 3DC2ME (Figure 5G–I). The nuclear translocation of NF-kB was also demonstrated by fluorescent immunostaining. The intensified nuclear translocation of NF-kB after treatment with 25 μM cisplatin visualized with a goat anti-rabbit IgG-heavy and light chain antibody FITC-conjugate (green) was reduced after pretreatment with 100 and 200 µM 3DC2ME (Figure 6).

## 4. Discussion

Cisplatin is known to lead to the death of kidney epithelial cells [15]., Our study demonstrated that treatment with 25 μM cisplatin for 24 h resulted in a decrease in cell viability and an increase in intracellular ROS in LLC-PK1 cells. By contrast, pretreatment with 3DC2ME significantly reduced the toxicity and intracellular levels of ROS. These results were consistent with the results of previous studies. Increased levels of intracellular ROS have been shown to play an important role in cisplatin-induced death of kidney epithelial cells, and this cell death was protected by pretreatment with antioxidants and ROS scavengers [16,17]. In addition, excessive levels of intracellular ROS have been linked to apoptotic cell death. In our previous study, 3DC2ME was shown to protect against cisplatin-induced apoptotic cell death [7]. In addition, a previous study showed that the protective effect of isoorientin against cisplatin-induced renal damage in mice was related to its antioxidant and anti-apoptotic activities [18]. Nrf2/HO-1 signaling has been shown to play an important role in the antioxidant mechanisms that control cisplatin-induced nephrotoxicity. Moreover, the transcription factor Nrf2 has been reported to regulate phase II detoxifying/antioxidant enzymes, including HO-1, superoxide dismutase, glutathione peroxidase, and catalase [19,20].

Previous studies have shown that the protective effects of melatonin [21], epigallocatechin-3-gallate [22], sinapic acid [23], and embelin [24] against cisplatin-induced renal damage in rats are related to antioxidant activity through the upregulation of Nrf2/HO-1 proteins. In LLC-PK1 cells, isoliquiritigenin exhibited protective effects against cisplatin-induced cell death by enhancing Nrf2 nuclear translocation and HO-1 [25]. In this study, pretreatment with 3DC2ME further intensified Nrf2 nuclear translocation and its downstream target, HO-1 protein expression. Referring to the results of previous studies, changes in these proteins may be part of the protective mechanism of 3DC2ME against cisplatin-induced cell death [21,22,23,24,25].

Oxidative stress enhances the secretion of pro-inflammatory cytokines, such as TNF-α, which amplifies the inflammatory response in cisplatin-induced renal damage [4]. TNF-α leads to the phosphorylation of IKKα/β, IκBα, and NF-κB [26]. Inflammatory mediators (iNOS and COX-2) at the gene transcriptional levels are regulated by the nuclear translocation of NF-κB [27]. MAPK (JNK and p38) indirectly regulates the nuclear translocation of NF-κB against cisplatin-induced nephrotoxicity in HK-2 human proximal tubular cells [28], rats [29], and mice [30]. In our previous study, 3DC2ME was shown to inhibit the expression of MAPK (JNK and p38) in cisplatin-treated LLC-PK1 cells [7]. Referring to the results of previous studies, JNK and p38 indirectly regulates the nuclear translocation of NF-κB in cisplatin-treated LLC-PK1 cells, which may also be part of the protective mechanism of 3DC2ME against cisplatin-induced cell death related to inflammation. Studies have reported that the administration of baicalein isolated from the roots of *Scutellaria baicalensis* Georgi [30] and saponins isolated from the roots of *Platycodon grandiflorum* reversed cisplatin-induced nephrotoxicity in mice by downregulating the expression of iNOS and COX-2 via the NF-κB pathway [31]. In line with previous studies, pretreatment with 3DC2ME inhibited the nuclear accumulation of NF-κB by downregulating the phosphorylation of IKKα/β and IκBα, thus suppressing the inflammatory response via the inhibition of iNOS and COX-2, which may also be part of the protective mechanism of 3DC2ME against cisplatin-induced cell death.

**Figure 7:**
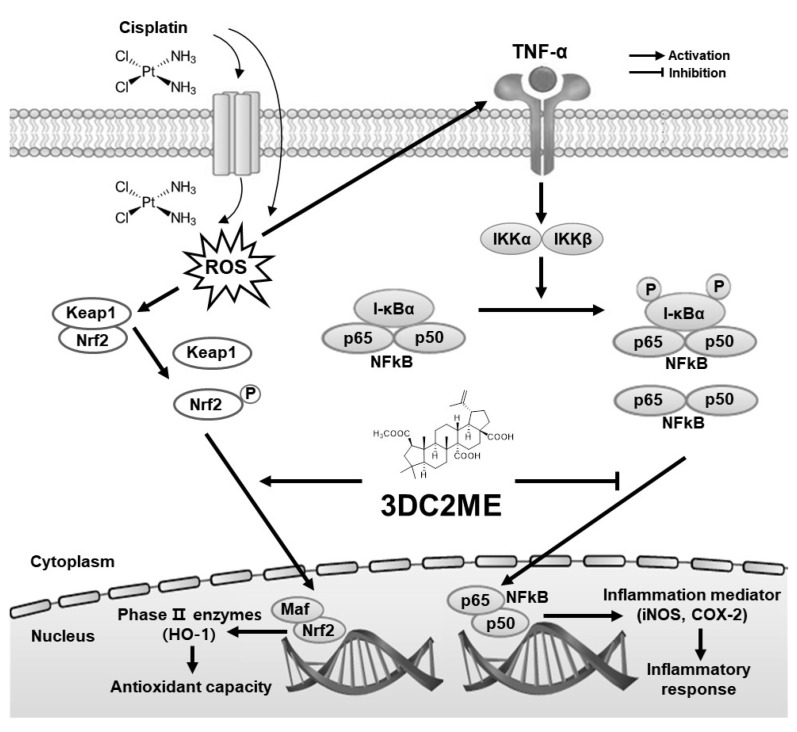
Schematic pathway for the potential mechanism of 3DC2ME in renoprotection.

In summary, our results demonstrated that 3DC2ME exerts a protective effect against cisplatin-induced cell death by suppressing the Nrf2/HO-1 mediated antioxidant pathway and NF-κB-mediated inflammation pathway (Figure 7). 3DC2ME could act as an antioxidant, anti-inflammatory, and nephroprotective agent in kidney epithelial cells challenged with cisplatin. Thus, our study suggests 3DC2ME as a potential candidate for the treatment of cisplatin-induced nephrotoxicity.

## 5. Conclusions

In this study, 3DC2ME isolated from *Z. jujuba* protected LLC-PK1 cells from cisplatin-induced cytotoxicity. 3DC2ME upregulated HO-1 via the Nrf2 pathway in cisplatin-treated LLC-PK1 cells. In addition, 3DC2ME inhibited the expression of IKKα/β, IκBα, NF-κB, iNOS, and COX-2 in the LLC-PK1 cells. Our results provide basic scientific evidence for the antioxidant and anti-inflammatory effects of 3DC2ME isolated from *Z. jujuba* against cisplatin-induced kidney epithelial cell death. Thus, our study suggests 3DC2ME as a potential candidate for the treatment of cisplatin-induced nephrotoxicity. However, further in vivo investigations and experiments using various kidney epithelial cell culture models are needed.

## Data Availability

Data is contained within the article or supplementary material.

## Supplementary Materials

The following are available online at https://www.mdpi.com/article/10.3390/biom11111614/s1, Figure S1: ^1^H NMR spectrum of 3DC2ME, Figure S2: ^13^C NMR spectrum of 3DC2ME, Figure S3: HRESIMS of 3DC2ME, Figure S4: HPLC-UV chromatogram of 3DC2ME.

## References

[1] Lopez-Giacoman S., Madero M. (2015). Biomarkers in chronic kidney disease, from kidney function to kidney damage. World J. Nephrol..

[2] Pabla N., Dong Z. (2008). Cisplatin nephrotoxicity: Mechanisms and renoprotective strategies. Kidney Int..

[3] Carreno J.J., Jaworski A., Kenney R.M., Davis S.L. (2013). Comparative incidence of nephrotoxicity by age group among adult patients receiving vancomycin. Infect. Dis. Ther..

[4] Ramesh G., Reeves W.B. (2005). p38 MAP kinase inhibition ameliorates cisplatin nephrotoxicity in mice. Am. J. Physiol. Ren. Physiol..

[5] Fang C.-Y., Lou D.-Y., Zhou L.-Q., Wang J.-C., Yang B., He Q.-J., Wang J.-J., Weng Q.-J. (2021). Natural products: Potential treatments for cisplatin-induced nephrotoxicity. Acta Pharmacol. Sin..

[6] Lee D., Choi S., Yamabe N., Kim K.H., Kang K.S. (2020). Recent findings on the mechanism of cisplatin-induced renal cytotoxicity and therapeutic potential of natural compounds. Nat. Prod. Sci..

[7] Lee D., Kim K.H., Lee W.Y., Kim C.-E., Sung S.H., Kang K.B., Kang K.S. (2019). Multiple targets of 3-dehydroxyceanothetric acid 2-methyl ester to protect against cisplatin-induced cytotoxicity in kidney epithelial LLC-PK1 cells. Molecules.

[8] Lee D., Kang K.B., Kim H.W., Park J.S., Hwang G.S., Kang K.S., Choi S., Yamabe N., Kim K.H. (2020). Unique triterpenoid of jujube root protects cisplatin-induced damage in kidney epithelial LLC-PK1 cells via autophagy regulation. Nutrients.

[9] Kandimalla R., Dash S., Kalita S., Choudhury B., Malampati S., Kalita K., Kalita B., Devi R., Kotoky J. (2016). Protective effect of bioactivity guided fractions of *Ziziphus jujuba* Mill. root bark against hepatic injury and chronic inflammation via inhibiting inflammatory markers and oxidative stress. Front. Pharmacol..

[10] Kandimalla R., Dash S., Kalita S., Choudhury B., Malampati S., Devi R., Ramanathan M., Talukdar N.C., Kotoky J. (2017). Bioactive fraction of *Annona reticulata* bark (or) *Ziziphus jujuba* root bark along with insulin attenuates painful diabetic neuropathy through inhibiting NF-κB inflammatory cascade. Front. Cell. Neurosci..

[11] Rahman M.A., Hwang H., Nah S.Y., Rhim H. (2020). Gintonin stimulates autophagic flux in primary cortical astrocytes. J. Ginseng Res..

[12] Yun M., Yi Y.S. (2020). Regulatory roles of ginseng on inflammatory caspases, executioners of inflammasome activation. J. Ginseng Res..

[13] Ryu Y.S., Hyun J.W., Chung H.S. (2020). Fucoidan induces apoptosis in A2058 cells through ROS-exposed activation of MAPKs signaling pathway. Nat. Prod. Sci..

[14] Lee S.R., Kang H., Yoo M.J., Yu J.S., Lee S., Yi S.A., Beemelmanns C., Lee J., Kim K.H. (2020). Anti-adipogenic pregnane steroid from a Hydractinia-associated fungus, *Cladosporium sphaerospermum* SW67. Nat. Prod. Sci..

[15] Kim Y.K., Kim H.J., Kwon C.H., Kim J.H., Woo J.S., Jung J.S., Kim J.M. (2005). Role of ERK activation in cisplatin-induced apoptosis in OK renal epithelial cells. J. Appl. Toxicol..

[16] Choi Y.-M., Kim H.-K., Shim W., Anwar M.A., Kwon J.-W., Kwon H.-K., Kim H.J., Jeong H., Kim H.M., Hwang D. (2015). Mechanism of cisplatin-induced cytotoxicity is correlated to impaired metabolism due to mitochondrial ROS generation. PLoS ONE.

[17] Gómez-Sierra T., Eugenio-Pérez D., Sánchez-Chinchillas A., Pedraza-Chaverri J. (2018). Role of food-derived antioxidants against cisplatin induced-nephrotoxicity. Food Chem. Toxicol..

[18] Fan X., Wei W., Huang J., Liu X., Ci X. (2020). Isoorientin attenuates cisplatin-induced nephrotoxicity through the inhibition of oxidative stress and apoptosis via activating the SIRT1/SIRT6/Nrf-2 pathway. Front. Pharmacol..

[19] Mahran Y.F. (2020). New insights into the protection of growth hormone in cisplatin-induced nephrotoxicity: The impact of IGF-1 on the Keap1-Nrf2/HO-1 signaling. Life Sci..

[20] Sahin K., Tuzcu M., Sahin N., Ali S., Kucuk O. (2010). Nrf2/HO-1 signaling pathway may be the prime target for chemoprevention of cisplatin-induced nephrotoxicity by lycopene. Food Chem. Toxicol..

[21] Ateşşahin A., Şahna E., Türk G., Çeribaşi A.O., Yılmaz S., Yüce A., Bulmuş Ö. (2006). Chemoprotective effect of melatonin against cisplatin-induced testicular toxicity in rats. J. Pineal Res..

[22] Sahin K., Tuzcu M., Gencoglu H., Dogukan A., Timurkan M., Sahin N., Aslan A., Kucuk O. (2010). Epigallocatechin-3-gallate activates Nrf2/HO-1 signaling pathway in cisplatin-induced nephrotoxicity in rats. Life Sci..

[23] Ansari M.A. (2017). Sinapic acid modulates Nrf2/HO-1 signaling pathway in cisplatin-induced nephrotoxicity in rats. Biomed. Pharmacother..

[24] Qin X., Meghana K., Sowjanya N.L., Sushma K.R., Krishna C.G., Manasa J., Sita G.J.A., Gowthami M., Honeyshmitha D., Srikanth G. (2019). Embelin attenuates cisplatin-induced nephrotoxicity: Involving inhibition of oxidative stress and inflammation in addition with activation of Nrf-/Ho-1 pathway. Biofactors.

[25] Moreno-Londoño A.P., Bello-Alvarez C., Pedraza-Chaverri J. (2017). Isoliquiritigenin pretreatment attenuates cisplatin induced proximal tubular cells (LLC-PK1) death and enhances the toxicity induced by this drug in bladder cancer T24 cell line. Food Chem. Toxicol..

[26] Huang N.-L., Chiang S.-H., Hsueh C.-H., Liang Y.-J., Chen Y.-J., Lai L.-P. (2009). Metformin inhibits TNF-α-induced IκB kinase phosphorylation, IκB-α degradation and IL-6 production in endothelial cells through PI3K-dependent AMPK phosphorylation. Int. J. Cardiol..

[27] Chiang Y.M., Lo C.P., Chen Y.P., Wang S.Y., Yang N.S., Kuo Y.H., Shyur L.F. (2005). Ethyl caffeate suppresses NF-κB activation and its downstream inflammatory mediators, iNOS, COX-2, and PGE2in vitro or in mouse skin. Br. J. Pharmacol..

[28] Ma X., Dang C., Kang H., Dai Z., Lin S., Guan H., Liu X., Wang X., Hui W. (2015). Saikosaponin-D reduces cisplatin-induced nephrotoxicity by repressing ROS-mediated activation of MAPK and NF-κB signalling pathways. Int. Immunopharmacol..

[29] Luo J., Tsuji T., Yasuda H., Sun Y., Fujigaki Y., Hishida A. (2008). The molecular mechanisms of the attenuation of cisplatin-induced acute renal failure by N-acetylcysteine in rats. Nephrol. Dial. Transplant..

[30] Wang Z., Sun W., Sun X., Wang Y., Zhou M. (2020). Kaempferol ameliorates Cisplatin induced nephrotoxicity by modulating oxidative stress, inflammation and apoptosis via ERK and NF-κB pathways. AMB Express.

[31] Zhang W., Hou J., Yan X., Leng J., Li R., Zhang J., Xing J., Chen C., Wang Z., Li W. (2018). Platycodon grandiflorum saponins ameliorate cisplatin-induced acute nephrotoxicity through the NF-κB-mediated inflammation and PI3K/Akt/apoptosis signaling pathways. Nutrients.

