# Supplementary material for: Antioxidant and Anti-Inflammatory Effects of 3-Dehydroxyceanothetric Acid 2-Methyl Ester Isolated from Ziziphus jujuba Mill. against Cisplatin-Induced Kidney Epithelial Cell Death
Source: Biomolecules. 2021 Oct 31;11(11):1614. doi: 10.3390/biom11111614 (PMC8615384; doi:10.3390/biom11111614)
Supplement: Supplementary file 1 [file biomolecules-11-01614-s001.zip › biomolecules-1430142-supplementary.pdf]

SUPPLEMENTARY DATA

Anti-oxidant and anti-inflammatory effects of 3-dehydroxyceanothetric acid 2-methyl ester isolated from *Ziziphus jujuba* Mill. against cisplatin-induced kidney epithelial cell death

Dahae Lee [1], Kyo Bin Kang [2], Gwi Seo Hwang [1], You-Kyoung Choi [1], Tae Kon Kim [3,*], and Ki Sung Kang [1,*]

[1] College of Korean Medicine, Gachon University, Seongnam 13120, Korea; pjsldh@gachon.ac.kr (D.L.); seoul@gachon.ac.kr (G.S.H.); kosmos@gachon.ac.kr (Y.-K.C.)

[2] College of Pharmacy, Sookmyung Women's University, Seoul 04310, Republic of Korea; kbkang@sookmyung.ac.kr (K.B.K.)

[3] College of Science & Engineering, Jungwon University, Chungbuk 28024, Republic of Korea

* Correspondence: kkang@gachon.ac.kr (K.S.K.), Tel +82-31-750-5402 (K.S.K.); tkkim@jwu.ac.kr (T.K.K.), Tel +82-43-830-8619 (T.K.K.)

Contents

Materials and Methods……………………………………………………….………3

Characterization data……………………………………………………………3

3-Dehydroxyceanothetric Acid 2-Methyl Ester

Figure S1. ^{1}H NMR spectrum of 3DC2ME..5

Figure S2. ^{13}C NMR spectrum of 3DC2ME..5

Figure S3. HRESIMS of 3DC2ME……………………………………………..6

Figure S3. HPLC-UV chromatogram of 3DC2ME…………………………………..6

Materials and Methods

Preparation of fractions and 3DC2ME from Ziziphus jujuba

The roots of *Z. jujuba* were collected in April 2012 at Jinju, Korea and authenticated by Prof. Dr. Eun Ju Jeong (Gyeongnam National University of Science and Technology, Jinju, Korea). A voucher specimen (SUPH-1204-01) was deposited in the Herbarium of the Medicinal Plant Garden, College of Pharmacy, Seoul National University, Koyang, Korea. Pulverized, air-dried roots of Z. jujuba (7.5 kg) were extracted with MeOH (2 × 30 L, for 3 h each) with ultrasonication at room temperature and then concentrated in vacuo. The crude extract (630.4 g) was suspended in H2O and partitioned successively into $CHCl_3$ (103.5 g), EtOAc (75.0 g), and BuOH fractions (127.3 g), respectively. The $CHCl_3$ fraction was subjected to silica gel CC eluted with mixtures of $CHCl_3$-MeOH (100:1, 50:1, 25:1, 15:1, 10:1, 7:1, 5:1, and 3:1) to yield ten fractions (C1–C10). The MeOH-soluble part of fraction C5 was subjected to silica gel CC eluted with $CHCl_3$-MeOH mixtures of increasing polarity (100:1, 50:1, 25:1, 15:1, 10:1) to give ten subfractions (C5a–C5j). Subfraction C5e was separated into seven further subfractions (C5e1–C5e7) by silica gel CC with mixtures of $CHCl_3$-MeOH of increasing polarity (100:1, 50:1, 25:1, 15:1, 10:1). White pellets of subfraction C5e2, which were insoluble in MeOH, were filtered and purified by recrystallization with MeOH to yield 3-dehydroxyceanothetric acid 2-methyl ester (3DC2ME) (45.8 mg).

Characterization data

3-Dehydroxyceanothetric Acid 2-Methyl Ester.
White amorphous powder; mp 296–298 °C; $[\alpha]_D^{20}$ +73.4 (c 0.10, MeOH); IR ν_{max} 3704, 2950, 2869, 2361, 2327, 1687, 1054, 1033, 1013 cm^{-1}; ^1H NMR (600 MHz, pyridine-d_5) δ 5.08 (s, 1H, H-29a), 4.82 (s, 1H, H-29b), 3.78 (s, -OCH_3), 3.71 (m, 1H, H-19), 2.98 (dt, 1H, *J* = 4.8, 12.9 Hz, H-13), 2.69 (d, 1H, *J* = 7.6 Hz), 1.92 (s, 3H, H-30), 1.17 (s, 3H, H-23),

1.16 (s, 3H, H-26), 0.89 (s, 3H, H-24), 0.89 (s, 3H, H-25); ^{13}C NMR (150 MHz, pyridine-d_5) δ 178.9 (C-27), 179.8 (C-28), 176.8 (C-2), 151.6 (C-20), 110.7 (C-29), 60.8 (C-14), 57.0 (C-17), 56.5 (C-5), 55.7 (C-1), 52.7 (C-18), 51.6 (C-10), 48. 3(C-19), 46.5 (C-9), 42.6 (C-3), 41.7 (C-8), 40.7 (C-13), 38.6 (C-4), 38.1 (C-22), 38.1 (C-7), 35.8 (C-16), 31.9 (C-23), 31.6 (C-21), 29.3 (C-15), 27.2 (C-12), 27.0 (C-24), 24.4 (C-11), 19.8 (C-25), 19.8 (C-30), 19.1 (C-6), 18.5 (C-26); HRESIMS *m/z* 513.3208 $[M - H]^-$ (calcd for $C_{31}H_{45}O_6$, 513.3216).

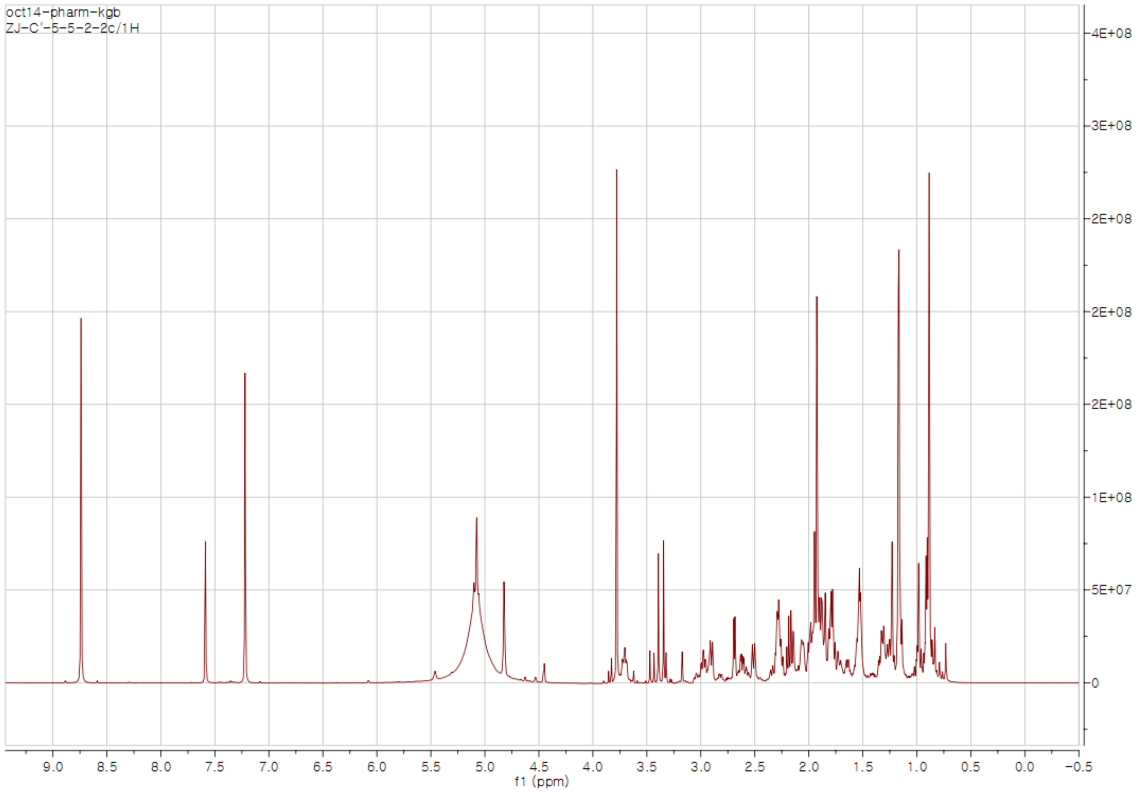

Figure S1. ^{1}H NMR spectrum of 3DC2ME.

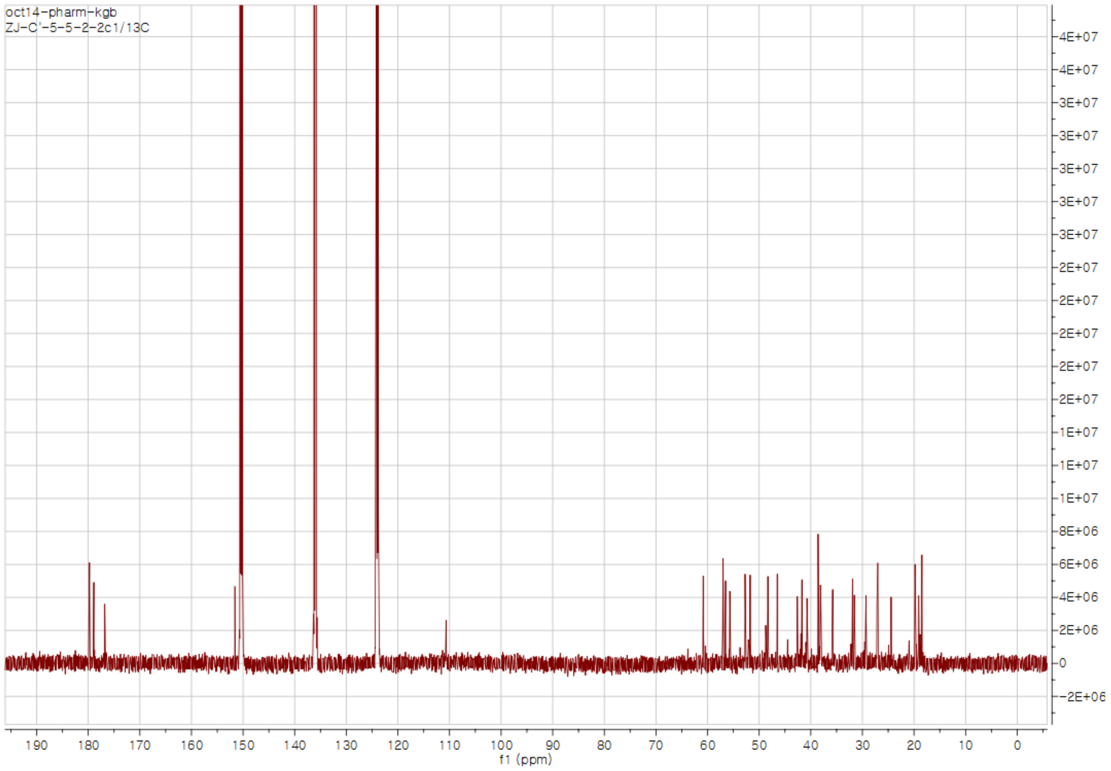

Figure S2. ^{13}C NMR spectrum of 3DC2ME.

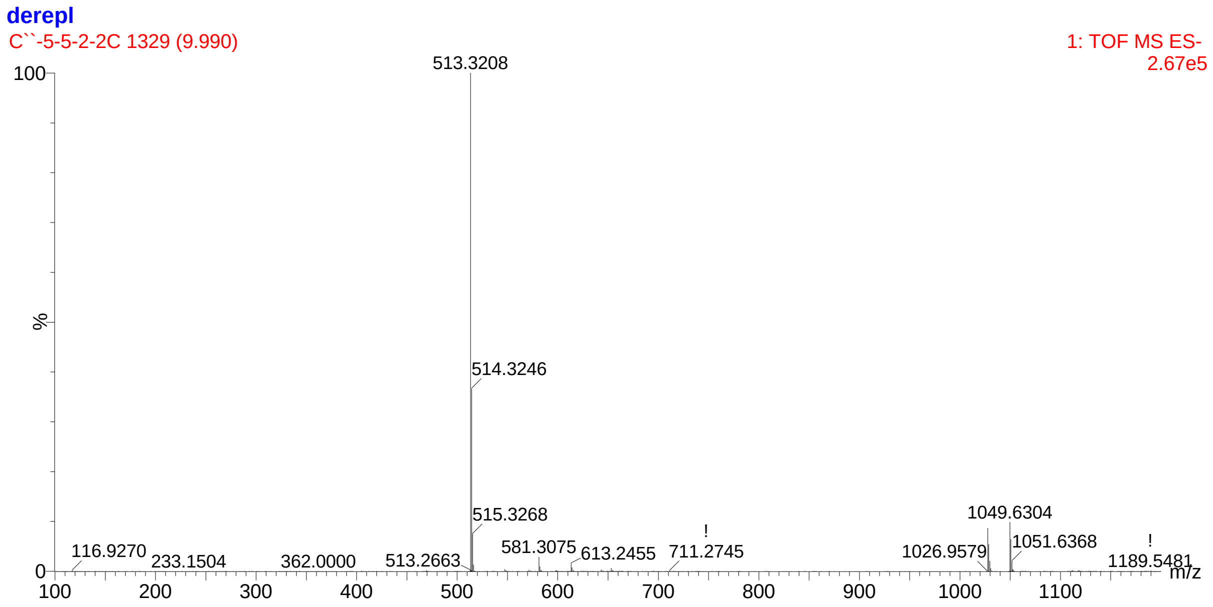

Figure S3. HRESIMS of 3DC2ME.

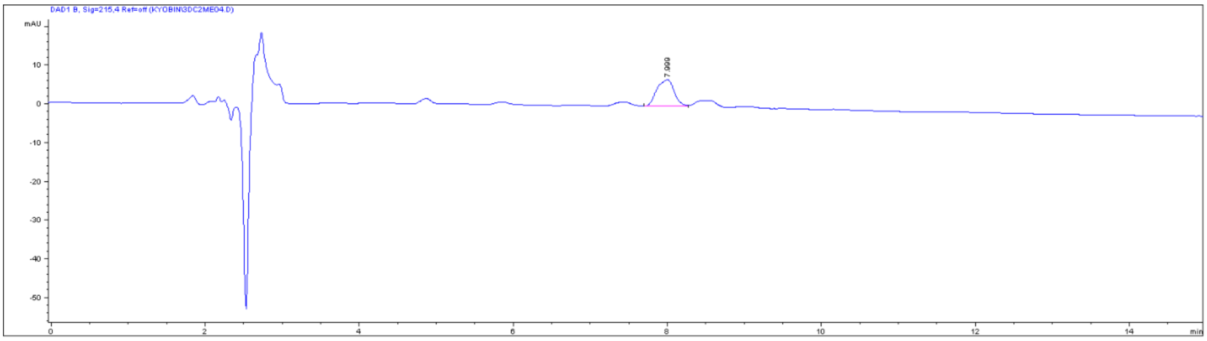

Figure S4. HPLC-UV chromatogram of 3DC2ME (82% MeCN at 1 mL/min, UV 215 nm).
